# Construct validity and factor structure of the Kessler-10 in South Africa

**DOI:** 10.1186/s40359-022-00883-9

**Published:** 2022-07-18

**Authors:** Jacob Hoffman, Qhama Cossie, Amantia A. Ametaj, Hannah H. Kim, Roxanne James, Rocky E. Stroud, Anne Stevenson, Zukiswa Zingela, Dan J. Stein, Bizu Gelaye

**Affiliations:** 1 Unit on Risk and Resilience in Mental Disorders, South African Medical Research Council (SAMRC), Cape Town, South Africa; 2 Department of Psychiatry and Mental Health, University of Cape Town, Cape Town, South Africa; 3 Neuroscience Institute, University of Cape Town, Cape Town, South Africa; 4 Department of Epidemiology, Harvard T.H. Chan School of Public Health, Boston, MA USA; 5 Department of Social and Behavioral Sciences, Harvard T.H. Chan School of Public Health, Boston, MA USA; 6 Psychology Department, Faculty of Health Sciences, Nelson Mandela University, Gqeberha, South Africa; 7 Stanley Center for Psychiatric Research at Broad Institute of MIT and Harvard, Cambridge, MA USA; 8 Department of Psychiatry, Harvard Medical School, Boston, MA USA

**Keywords:** Factor analysis, statistical, Prevalence, Psychological distress, Psychometrics, South Africa, Theoretical models, Validity and reliability

## Abstract

**Background:**

The Kessler Psychological Distress Scale (K-10) is a short screening tool developed to identify, with good sensitivity, non-specific psychological distress in the general population. Sensitivity and specificity of the K-10 have been examined in various clinical populations in South Africa; however, other psychometric properties, such as construct validity and factor structure, have not been evaluated. We present evidence of the prevalence and severity of psychological distress in an outpatient setting in South Africa and evaluate the internal reliability, construct validity, and factor structure of the K-10 in this population.

**Methods:**

We explored prevalence estimates of psychological distress using previously established cutoffs and assessed the reliability (consistency) of the K-10 by calculating Cronbach’s alpha, item-total correlations and omega total and hierarchical coefficients. Construct validity and factor structure of the K-10 were examined through split-sample exploratory factor analysis (EFA) followed by confirmatory factor analysis (CFA), comparing several theoretical models and the EFA.

**Results:**

Overall, there was low prevalence of psychological distress in our sample of 2591 adults, the majority of whom were between the ages of 18–44 (77.7%). The K-10 showed good construct validity and reliability, with a Cronbach’s alpha of 0.84 and omega total of 0.88. EFA yielded a four-factor solution with likely measurement artifacts. CFA showed that the four-factor model from EFA displayed the best comparative fit indices, but was likely overfitted. The unidimensional model with correlated errors was deemed the best fitting model based on fit indices, prior theory, and previous studies.

**Conclusion:**

The K-10 displays adequate psychometric properties, good internal reliability, and good fit with a unidimensional-factor structure with correlated errors. Further work is required to determine appropriate cutoff values in different populations and clinical subgroups within South Africa to aid in determining the K-10’s clinical utility.

## Background

Common mental disorders, such as mood and anxiety disorders, contribute significantly to the global disease burden [1]. Few individuals with mood and anxiety disorders receive the treatment that they need, and this is particularly true in low- and middle-income countries (LMICs) [2]. A first step toward closing this gap in care may be to use population-level studies to determine the prevalence of common mental disorders, which can help develop public health policies and plans for treatment funding [3].

Additionally, effective screening tools such as the Kessler Psychological Distress Scale (K-10) may be used in a clinical context to identify individuals at risk of mental illness and to improve treatment rates. Yet, many assessment tools have been developed in well-resourced settings and require validation within LMICs where there are significant financial and human resource constraints, including a relative lack of trained mental health care personnel [4]. Having access to validated, lay-administered tools that are easy and quick to administer make nationwide epidemiological studies more feasible [3], improving efforts to monitor and treat common mental disorders [5].

The K-10 has been used as a screening tool for mood and anxiety disorders and was specifically developed for population-based studies to identify individuals experiencing non-specific psychological distress and screen for symptoms of anxiety and depression [6]. The K-10 has shown good sensitivity and specificity for predicting mental disorders defined by the Diagnostic and Statistical Manual of Mental Disorders-5 (DSM-5; [5], as measured by the World Health Organization Composite International Diagnostic Interview (CIDI; [3, 6]. In recent years, the K-10 has gained popularity, and several studies have investigated its psychometric properties [3, 7], including in low-resource settings [3, 8–11]. Translated versions of the ten-item scale (K-10) and the abbreviated six-item scale (K-6) have strong psychometric properties in Vietnamese [12], Dutch [13], Arabic [13, 14], and Turkish language [13] with generally good discriminating ability between non-cases and cases with mental disorders as defined by the DSM-5 [5] and measured using the CIDI [3]. Studies examining the factor structure of the K-10 among various populations have found a unidimensional factor [12, 13, 15] and a unidimensional factor with correlated errors [15] for data from community-based populations, and a two-factor model with anxiety and depression symptoms converging onto two separate groupings with clinical populations with mental health conditions [15].

In South Africa, a LMIC, psychometric properties of the K-10 and abbreviated K-6 have been explored for specificity, internal consistency and sensitivity in several studies within a representative South African sample and a few clinical populations [9–11, 16, 17]. For example, K-10 was investigated in a nationally representative household survey of 4077 adults and demonstrated moderate discriminating ability in detecting mood and anxiety disorders compared with the CIDI; however, its sensitivity and specificity were determined to be inadequate within the South African context [9]. Therefore, further work is required to determine whether the K-10 could be feasibly used within South Africa in a cost-effective manner that would improve detection rates of common mental disorders in the country. In addition, other psychometric properties such as factor structure (i.e., the relationship between items testing the same construct) and construct validity (i.e., whether the test measures what it intends to) of the K-10 have not yet been investigated in South Africa. These are important to determine a screening tool’s validity when applied to a new population. Lastly, a few studies have investigated the prevalence of psychological distress in primary care outpatient settings in South Africa and other LMICs [10, 11, 16, 18], and the relatively high prevalence of psychological distress in these studies indicate the need for further research into screening tools such as the K-10 [11, 16, 18].

In African countries, only a few studies have investigated the factor structure of the K-10. A study in Tanzania in a clinical sample of individuals with traumatic brain injury showed acceptable psychometric properties and adequate support for both a unidimensional model of psychological distress and a two-factor model of depression and anxiety [8]. In an Ethiopian community sample, a two-factor model was derived from exploratory factor analysis (EFA) [19]. More recently, our colleagues in Ethiopia and Kenya examined the K-10 factor structure using the same study design we propose here. In Ethiopia, a two factor model emerged from the EFA and a unidimensional model with correlated errors was ultimately the best fitting model [18]. Similarly, in the sample from Kenya, a two-factor model was derived from EFA, however a unidimensional model with correlated errors again showed the best fit [20].

The objectives of the current study are to estimate the prevalence of psychological distress in South Africa and further investigate the psychometric properties of the K-10 in a sample of adults in outpatient settings in Western and Eastern Cape. We examined the reliability of the measure in South Africa with our population, and an EFA and confirmatory factor analysis (CFA) based on prior studies [12, 13, 15] were used to examine the construct validity, and factor structure of the K-10 in this context.

## Methods

### Study setting and sample

Data were derived from South Africa as part of the Neuropsychiatric Genetics of African Populations-Psychosis (NeuroGAP-Psychosis) study, an ongoing multi-country case–control and genome-wide association study (GWAS) to deepen understanding of genetic and environmental risk factors for psychotic disorders in Africa [21]. A full explanation of the methodology of the NeuroGAP-Psychosis study is detailed elsewhere [21]. Participants enrolled in NeuroGAP-Psychosis in South Africa were recruited starting in April 2018, and we restricted our analysis to data from South Africa through the end of March 2020. Participants were recruited from the following medical facilities: Fort England Psychiatric Hospital and satellite clinics (Grahamstown, Eastern Cape), Nelson Mandela Academic Hospital (Mthatha, Eastern Cape), Valkenberg Hospital (Cape Town, Western Cape), and several community clinics in the Cape Town Metropolitan Area. Findings for this study were limited to control participants from the parent study because the K-10 was administered only to individuals who served as controls in the NeuroGAP-Psychosis study. Control participants include individuals seeking clinical care for themselves at outpatient general medical facilities, those accompanying a friend or family member to a clinic visit, workers at the hospital/clinic, or those attending for any other reason, such as collecting a medication refill. Inclusion criteria for control participants were being from the same geographic location as NeuroGAP-Psychosis case participants but without a clinical diagnosis of psychosis and not taking psychotropic medication [21]. Exclusion criteria consisted of currently having psychotic symptoms or a past diagnosis of a psychotic disorder, currently taking medication for psychosis, undergoing treatment for alcohol or substance misuse (i.e., current inpatient or currently under acute medical care for substance misuse), or lacking the capacity to consent to the study, as determined by the University of California, San Diego Brief Assessment of Capacity to Consent [21].

Ethical approval to conduct this study was obtained from all participating sites, including the University of Cape Town Human Research Ethics Committee (REF# 466/2016), the Western Cape Government (WC_2016RP32_349), and the Walter Sisulu University Research and Ethics Committee (SOMREC #REC REF 2016-057) in South Africa and the Harvard T.H. Chan School of Public Health (#IRB17-0822) in the United States. All study protocols were approved by the above-named institutions and/or ethics committees. Informed consent was obtained from all study participants and all study procedures were conducted in accordance with the relevant guidelines and regulations.

### Measures

#### K-10

The K-10 is a ten-item questionnaire that assesses the presence of general psychological distress experienced in the four weeks prior to administration [6]. Individual items assess symptoms commonly associated with depression and anxiety, and each is assessed on a five-point scale from 0 to 4, with increasing values corresponding to higher levels of distress. A total score is calculated by summing all items, ranging 0–40. Items are introduced with the statement, “The following questions ask about how you have been feeling during the past 30 days. For each question, please identify the best answer that describes how often you had this feeling.” Prior studies in the South African context have found cutoff values of 6 [9], 11.5 [10], and 18 [11] for current mood or anxiety disorder, and the scale has shown good internal consistency (Cronbach’s α = 0.84 [9], α = 0.87 [11], α = 0.89 [16], and α = 0.92 [17]). Cutoff values may vary based on the specific patient population being studied and may be further adjusted to balance sensitivity and specificity within the patient population in question. Other studies report brackets of total K-10 scores to discriminate between different severity levels of psychological distress, from mild to severe [16]. The above studies scored the K-10 from 10–50; therefore, reported cutoffs in this paper have been adapted to be consistent with a scale of 0–40.

#### Demographic characteristics

All participants enrolled in the study provided information on several demographic variables, including age, level of education, marital status, current living situation, and sex at birth. This was collected using encrypted tablets and uploaded to a secure cloud-based server.

### Statistical analysis

We first used descriptive analyses [means and standard deviations (SDs) for continuous variables, and counts and percentages for categorical variables] to characterize the study sample. Next, we explored prevalence estimates of psychological distress using previously established cutoffs from other studies in South Africa and elsewhere [7, 16]. We assessed reliability (consistency) of the K-10 by calculating Cronbach’s alpha (> 0.8 considered acceptable) [22] and item-total correlations (> 0.2 considered significant)[23]. The coefficient omega total and coefficient omega hierarchical were also calculated, which are used to assess reliability of the general factor in a bifactor model [24]. In addition, we examined an aspect of construct validity through the factor structure of the K-10 by conducting a random split-sample EFA followed by CFA exploring several theoretical models and the EFA from the other split sample. CFA for the model derived from the EFA was conducted on the other half of the split-sample, whilst CFA for the other theoretical models was conducted on the full sample.

Before performing EFA, we assessed suitability of the data for performing factor analysis by calculating Bartlett’s test of sphericity [25] and the Kaiser–Meyer–Olkin measure of sampling adequacy [26]. We conducted the EFA extracted factors using a principal axis factoring with oblique rotation, which assumes correlation of variables. We used parallel analysis for polychoric factors with 50 iterations to investigate dimensionality. The number of factors retained for rotation was determined by the intersection point of the actual data plot with the simulated data plot [27]. Rotated factor loadings of > 0.3 were considered sufficient, while items with factor loadings ≥ 0.3 on more than one factor were considered cross-loading. For cross-loading items, the highest factor loading with the strongest correlation was used when assigning them to a single factor.

In the CFA, we examined results for the following four models: (1) a unidimensional model, (2) a unidimensional model with correlated errors, (3) a two-factor model with depression and anxiety as latent variables (similar to Sunderland, Mahoney, & Andrews, 2013) [15], and (4) this study’s EFA. To compare the four CFA models, we used the following metrics of model fit: a root mean square error of approximation (RMSEA) of ≤ 0.06, with a lower score indicating a better fit; a comparative fit index (CFI) of ≥ 0.90, with a higher score indicating a better fit; and a Tucker–Lewis index (TLI) of ≥ 0.90, with a higher score indicating a better fit [29, 30]. Models with scores that did not meet these thresholds were considered inadequate. Path diagrams were generated for graphical representation of factor loadings in each model. All statistical analyses were performed using Stata 15 [31]. All *P* values were two-sided and set at an alpha level of 0.05. CFA was conducted using diagonally weighted least squares (DWLS) to estimate the model parameters, and the full weight matrix used to compute robust standard errors, and a mean- and variance-adjusted test statistic.

## Results

There were 2591 participants included in the analysis, most recruited from community clinics in Cape Town, Western Cape (n = 2423, 93.5%). The other 6.5% of participants were recruited from outpatient settings in other South African cities in the Eastern Cape, including Mthatha (n = 57) and Grahamstown (n = 95). Roughly half of participants were female (51.6%) and single (55.4%) (Table 1). A majority of participants were 18–44 years old (77.7%) and had at least some secondary level of education (91.2%).

**Table 1:** Participant demographics of South African sample population (N = 2591)

	Count	%
*Sex (%)*		
Female	1337	51.6
Male	1254	48.4
Age (median, IQR)	33.0	26–43
*Age categories (%)*		
18–29	955	36.9
30–44	1056	40.8
45–59	493	19.0
≥ 60	87	3.4
*Marital status (%)*		
Single	1436	55.4
Married or cohabitating	880	34.0
Widowed	64	2.5
Divorced or separated	204	7.9
*Level of education (%)*		
No formal	8	0.3
Primary	218	8.4
Secondary	1877	72.4
University	486	18.8
*Living arrangements (%)*		
Lives alone	610	23.5
Lives with parental family	630	24.3
Lives with spouse or partner	875	33.8
Lives with friends or other relatives	453	17.5

Counts may not add up to total due to missing information for some categories from some participants

*IQR* interquartile range

**Fig. 1:**
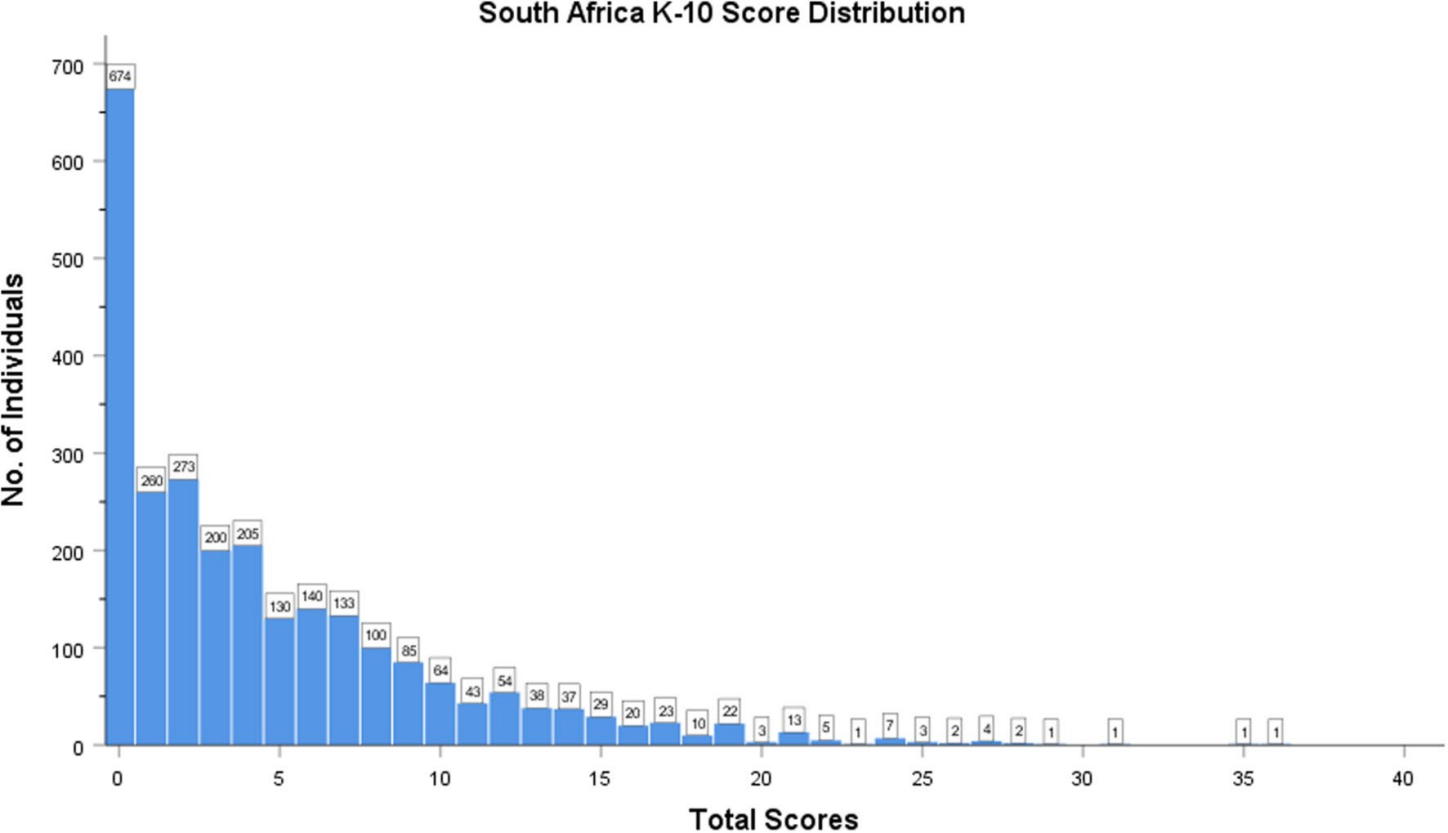
Distribution of global K-10 scores. Scores are presented as the sum of all answers. Higher scores represent a higher likelihood of having a mental health disorder

Scores for individual items were summed to give each participant a score out of 40, the distribution of which is represented in Fig. 1. A higher score indicates a greater likelihood of having psychological distress. We calculated prevalence proportions based on cut-off scores from prior studies of K-10 in South Africa and elsewhere [7, 16]. Only 1.7% of participants were likely to have a severe mental disorder based on a cutoff of 20 (n = 44). Using alternate cutoff scores of 6 and 10, the frequency of psychological distress was 32.6% (n = 842) and 14.9% (n = 384), respectively, and 85.1% (n = 2200) had a score of < 10.

Mean raw scores for each item were low across all items (Table 2). “Fatigue” had the highest mean score (0.86, SD = 1.09), and “so depressed” had the lowest mean score (0.13, SD = 0.51). Internal consistency was tested by calculating Cronbach’s alpha (⍺ = 0.84), which indicated good internal consistency (reliability) for the K-10 scale with this population. Removal of any question from the K-10 scale resulted in a lower Cronbach’s alpha, indicating that all items are worthy of retention. The coefficient omega hierarchical was calculated as 0.68 across all variables, and the omega total was 0.88. While there is no universally accepted guideline for adequate levels of omega reliability, omega hierarchical and omega total coefficients should exceed 0.50 at a minimum and values closer to 0.75 would be preferred [32].

**Table 2:** Item characteristics, item-total correlations, and ⍺ if item deleted from the K-10

K-10 items	Mean	SD	Corrected item-total correlation	⍺ if item deleted
Fatigue	0.86	1.09	0.49	0.83
Nervous	0.67	0.92	0.56	0.82
So nervous	0.20	0.58	0.46	0.83
Hopeless	0.67	0.95	0.53	0.82
Restless fidgety	0.44	0.81	0.54	0.82
So restless	0.18	0.55	0.50	0.82
Depressed	0.38	0.81	0.58	0.81
So depressed	0.13	0.51	0.53	0.82
Lack of energy	0.58	0.92	0.60	0.81
Worthless	0.45	0.82	0.60	0.81
Global K-10 score^a^	4.56	5.18	–	0.84

^a^Overall Cronbach’s alpha

### EFA

**Fig. 2:**
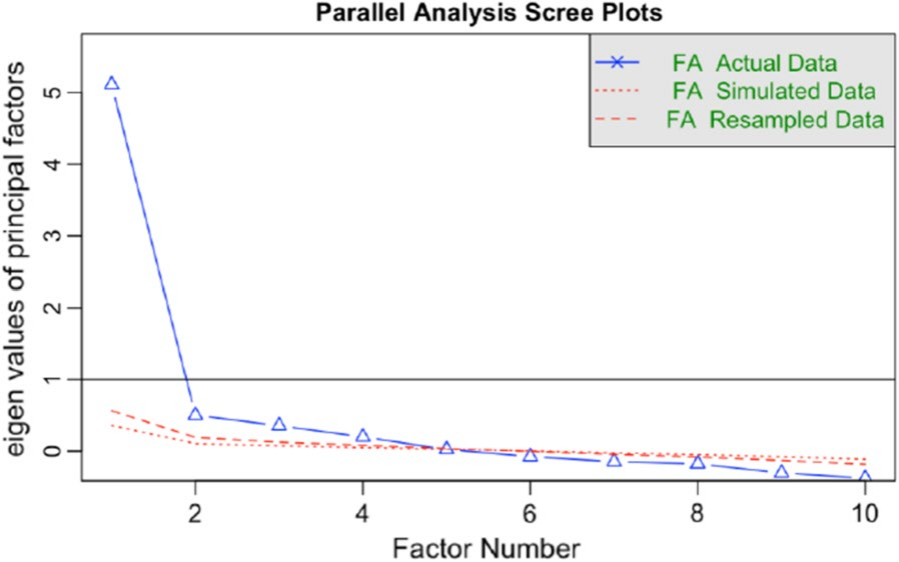
Parallel analysis scree plot of the K-10. The number of factors retained from visual interpretation is 4 factors for EFA

Analyses testing suitability of the data showed that it was appropriate to proceed with factor analysis (Bartlett’s test of sphericity, χ^2^(45) = 4168.93; P < 0.001; Kaiser–Meyer–Olkin measure of sampling adequacy = 0.83). To conduct the EFA, the sample was randomly split into two datasets. Using EFA in the first subsample (N = 1295), we examined the data to assess scale dimensionality and item-factor loadings. Parallel analysis for polychoric factors with 50 iterations suggests four factors (Fig. 2).

Results of the EFA using an oblique rotation indicated a four-factor solution (Table 3). Two items, “depressed” (r = 1.024) and “so depressed” (r = 0.685) loaded on factor 1. Three items, “fatigue” (r = 0.379), “nervous” (r = 0.618) and “so nervous” (r = 0.985) loaded on factor 2. Two items, “restless” (r = 0.889) and “so restless” (r = 0.891) loaded on factor 3. The last three items, “lack of energy” (r = 0.501), “worthless” (r = 0.866) and “hopeless” (r = 0.744) loaded on factor 4. The four factors derived from EFA explained 63.9% of the total variance. Inter-factor correlations between factors 1 and 2 was 0.50, between factors 1 and 3 was 0.56, between factors 1 and 4 was 0.66, between factors 2 and 3 was 0.63, between factors 2 and 4 was 0.74 and between factors 3 and 4 was 0.61. There were no cross-loading items. Factor loadings for factors 1 and 3 appear to be quite high (e.g., “depressed” loading exceeds 1 at 1.024). Overall, this model does not appear to be parsimonious given that two factors have only two items each, and items seem to be grouped based on similar wording ("depressed” and “so depressed” on factor 1 and “restless” and “so restless” on factor 3).

**Table 3:** Factor loadings with oblique rotation for EFA of K-10

	Factor 1	Factor 2	Factor 3	Factor 4
Fatigue		0.379		
Nervous		0.618		
So nervous		0.985		
Restless/fidgety			0.889	
So restless			0.891	
Depressed	1.024			
So depressed	0.685			
Lack of energy				0.501
Worthless				0.866
Hopeless				0.744
SS Loadings	1.574	1.568	1.612	1.641
Proportion Variance	0.157	0.157	0.161	0.164
Cumulative Variance	15.7%	31.4%	47.5%	63.9%

Standardized loadings using factor analysis with oblique rotation for the K-10 (retained factors = 4) for the split sample (n = 1295) for the EFA. ^1^Bartlett test of sphericity, χ^2^(45) = 4168.93; P < 0.001; Kaiser–Meyer–Olkin measure of sampling adequacy = 0.83. Any loadings < 0.30 were dropped

### CFA

**Fig. 3:**
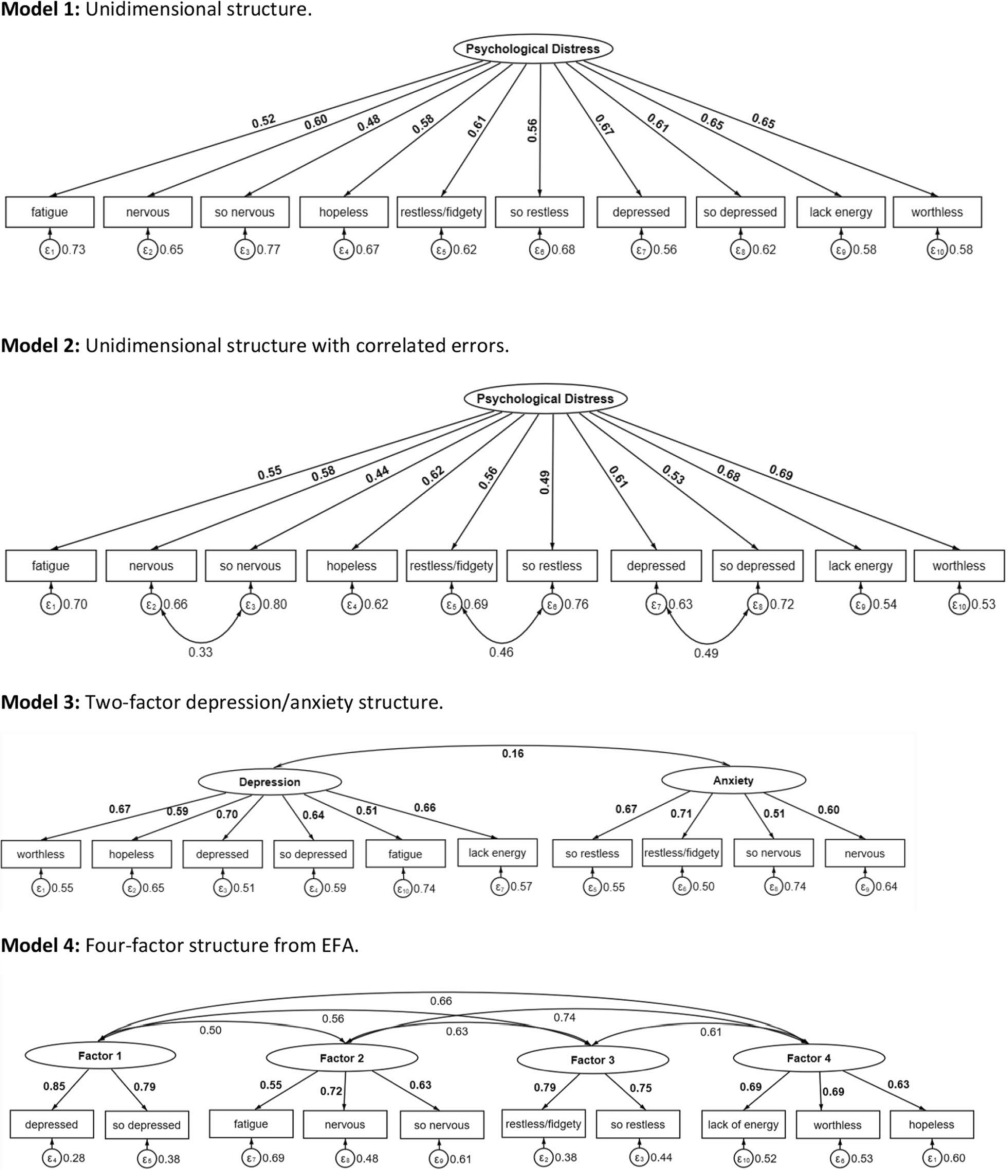
Path diagrams of the three tested theoretical structure models for the K-10 (models 1–3) and the model derived from EFA (model 4). Factor loadings are standardized estimates

We estimated three theoretical models informed by the literature and one model informed by the EFA. The first three theoretical models were applied to the entire dataset (N = 2591). The other random split-half sample (n = 1296) was used to test the factor structures of the fourth model derived from the EFA. These models and respective factor loadings with errors are graphically represented as path diagrams (Fig. 3).

The four models were tested for comparative fit (Table 4). Factor loadings for all models (as seen in Fig. 3) were acceptable and above the 0.4 cut-off. Model 4 derived from the EFA and tested using the other half of the split-sample showed the best fit indices (CFI = 0.97, TLI = 0.95, RMSEA = 0.06). A unidimensional model with correlated errors (Model 2) showed the second-best fit (CFI = 0.95, TLI = 0.93, RMSEA = 0.07). This model was composed of one factor, psychological distress, but incorporated correlated errors between the following pairs: “depressed” and “so depressed,” “restless” and “so restless,” “nervous” and “so nervous.” The other two models performed poorly across all fit indices, with CFI ≤ 0.90, TLI ≤ 0.90, and RMSEA ≥ 0.10. Model 1 was composed of one factor, psychological distress, loading onto all K-10 items. This model had poor overall fit (CFI = 0.79, TLI = 0.72, RMSEA = 0.14). Model 3 was composed of two factors, depression, and anxiety, and displayed poor fit overall (CFI = 0.82, TLI = 0.76, RMSEA = 0.13).

**Table 4:** Confirmatory factor analysis fit statistics for each of the four models

Model	χ^2^	df	*P*	CFI	TLI	RMSEA
Model 1	1826.57	35	< 0.001	0.79	0.72	0.14
Model 2	463.45	32	< 0.001	0.95	0.93	0.07
Model 3	1577.63	34	< 0.001	0.82	0.76	0.13
Model 4	166.99	29	< 0.001	0.97	0.95	0.06

Model 1, unidimensional structure. Model 2, unidimensional structure with correlated errors. Model 3, two-factor depression/anxiety structure. Model 4, four-factor model from EFA. Abbreviations: CFI, comparative fit index; χ2, chi-square statistic; df, degree of freedom; P, significance level; RMSEA, root mean square error of approximation; TLI, Tucker–Lewis fit index

## Discussion

Our main findings were that: (1) there were low levels of non-specific psychological distress in a South African urban and peri-urban outpatient setting, (2) the K-10 displayed good construct validity and reliability, and (3) a unidimensional model with correlated errors was the best-fitting model in our population based on both prior theory and research evidence and as indicated by the model fit indices. A four-factor model (derived from the EFA analysis of our data) displayed the best fit indices comparatively but produced a model solution that was likely due to methodological artifacts with similarly worded items converging together.

Overall, there were low levels of psychological distress in our sample. Most participants (85.1%) obtained a K-10 score less than 10, and only 1.7% of participants had a score higher than 20, which has been reported as a cutoff for severe psychological distress in South Africa [16]. We did not identify a cut-off score for our sample because we lacked a measure for criterion validity. However, we calculated prevalence proportions based on cut-off scores from prior studies in South Africa [7, 16]. Our findings showed lower levels of psychological distress compared with prior research in South Africa [11, 16, 17, 33, 34]. A previous study in a South African outpatient hospital demonstrated no significant distress in only 50.3% of participants [16]. The other 49.7% of participants scored greater than 10, and displayed higher levels of psychological distress compared to our study, with 17.1% of participants with scores greater than 20. Similar high levels were found in other clinical groups in South Africa, such as patients with tuberculosis [17], HIV-positive individuals [11], and antenatal women [10, 35]. The differences in overall levels of psychological distress in different clinical populations in South Africa may be due to medical condition-specific factors, or may be reflective of regional socioeconomic variability across South Africa [36]. Most of our sample was recruited from Cape Town and surrounding areas. On the other hand, our findings are similar to the prevalence of mild or moderate psychological distress with cutoff scores of 6 (32.5%) or 10 (14.8%) in other population-based studies in Australia [7]. Prevalence of low (85.1%) and severe (1.7%) levels of psychological distress in our sample is therefore consistent with non-clinical samples and is lower than clinical samples, which may be expected as our study was conducted among a diverse group of participants in outpatient settings. These include people seeking healthcare for themselves, caregivers bringing a friend or family member to a clinic, workers at a hospital/clinic, and people getting a prescription refill.

Regarding its psychometric properties, the K-10 demonstrated good construct validity and reliability when used among South African adults attending outpatient clinics. The overall Cronbach’s alpha was 0.84, which is consistent with other studies both in South Africa and other settings [6, 8, 9, 11, 14, 17, 35, 37]. In addition, the coefficient omega hierarchical was 0.68 across all variables, and the omega total was 0.88, both of which are close to the recommended 0.75 [32]. The EFA yielded a four-factor solution; this model produced the best-fitting indices across CFAs. However, inferences from the four-factor model in our study is limited as the model has fewer degrees of freedom and two of the factors have only two items with similar wording (i.e., “restless” and “so restless” on one factor and “depressed” and “so depressed” on another factor). Next to this four-factor model, the best-fitting model following CFA was a unidimensional model with correlated errors, which is also consistent with previous studies that showed a unidimensional model to be the best fit [3, 6, 15].

The K-10 was originally reported to have a unidimensional factor structure [6], similar to the abbreviated K-6 [3]. However, multidimensional factor structures have been suggested in several studies, including with a group of post-natal women in Ethiopia and a sample of patients with traumatic brain injury in Tanzania [8, 14, 15, 28, 37, 38]. However, this is the first investigation of the factor structure of the K-10 in South Africa. EFA yielded a four-factor structure, but the individual factor loadings were not consistent with previous multidimensional models derived from EFA in other countries [14, 15, 28, 38]. Overall, differences in factor structures across studies may be due to differences in cultural interpretation of depression and anxiety symptoms. Additionally, the variance explained by the four-factor model was only 63.9%. The low variance found in our study is indicative that the items on the K-10 may not be sufficiently explaining the model. Additional items incorporating local expressions of distress might help clarify the factor structures and improve the variance in the screening measure.

Multidimensional factor structures typically report an anxiety factor and a depression factor for two-dimensional models; however, these may be subdivided into a second-order factor structure wherein second-order anxiety is represented by a nervous factor and an agitation factor, and second-order depression is represented by a fatigue factor and a negative affect factor [38]. This is similar to the pattern of factor loadings in other two-factor models [14, 15, 28].

In our study, the pattern of association of individual items with either anxiety or depression was different. “Depressed” and “so depressed” loaded on a single factor representing depression. “Restless” and “so restless” also loaded on a single factor. “Nervous,” “so nervous” and “fatigue” loaded on a third factor, which may represent anxiety. Finally, “lack of energy,” “worthless” and “hopeless” loaded on a fourth factor. This combination differs from previous reports as it combines nervousness with fatigue rather than restlessness and shows a dissociation of psychological nervousness from physical restlessness in the factor loadings. Also, symptoms traditionally linked to depression such as lack of energy, worthlessness and hopelessness were loaded on a separate factor rather than being associated with the depression factor as reported in other studies [14, 15, 28, 38]. Though it is possible that these differences are due to cultural interpretations, translations, expression, or experience of depression and anxiety and their associated features within our study sample; a more likely interpretation is that the EFA represents measurement artifacts given that similarly worded items were grouped together.

While EFA yielded a four-factor structure, and CFA confirmed this to be the best-fitting model, a one-factor solution of non-specific psychological distress with correlated errors also showed good results with regards to fit indices and is consistent with prior studies [3, 6, 20, 39]. A unidimensional model with correlated errors appears to be a more appropriate model given its adequacy of fit and correlation with previous theory and research [3, 6, 15]. Studies in other countries have reported similar RMSEA values for a unidimensional model [15, 38], although multifactorial models have shown better fit in other countries, particularly in Australia [38, 40] and specifically in clinical populations [15]. Both unidimensional models and multidimensional models for the K-10 have shown adequate fit in other LMICs, such as Kenya, Tanzania, Ethiopia, and West Bank territory [8, 14, 20, 37, 39]. Next to the four-factor solution, our findings suggest a unidimensional model for the K-10 in South Africa, which is consistent with work on the K-6 [3], and helps confirm its construct validity within this context. The lack of prior, local studies investigating factor structure of the K-10 for comparison limits the contextual interpretation of our results.

## Future directions and limitations

Previous studies in South Africa have been unable to determine a cutoff value for psychological distress that optimally balances sensitivity, specificity, and positive predictive value (PPV) for the K-10 [9]. These should be balanced, particularly in resource-constrained settings, to avoid misallocation of resources for false positives, ensure a low false negative rate, and reduce the likelihood of missing treatable psychopathology [35]. Future studies comparing the K-10 with a gold-standard diagnostic instrument for psychopathology, such as the CIDI, would help to establish cutoff scores for the K-10 in South Africa that optimally balance sensitivity, specificity, and PPV. An option in future studies could be to report a range of cutoff values, which would aid in interpreting the results, stratifying findings based on severity, and informing future cutoff scores in validation studies and may be clinically useful [41].

Findings from this study should be understood within its limitations. We report on factorial validity of K-10, which is one aspect of construct validity. Research on other methods to measure construct validity may also be needed to replicate our findings. In addition, other aspects of validity including convergent and criterion validity are not addressed and should be examined in future studies. Our study sample consisted of a diverse group of individuals as control participants, both healthcare seeking-populations and non-healthcare seeking-populations in a larger study, who may vary from the general population in unknown ways and may limit the generalizability of our findings. Despite these limitations, our study consisted of a large sample size and is the first of its nature to examine factor structure of K-10 in South Africa. Areas for further research include investigating differences between subgroups, including the presence or absence of medical comorbidities, recent potentially traumatic events, and language and ethnicity differences. Future studies may also compare the K-10 with gold-standard diagnostic instruments to further investigate criterion validity, including sensitivity and specificity of the K-10 in South Africa, and convergent validity by comparison with other tests.

## Conclusion

The K-10 was appropriate for use as a tool to measure non-specific psychological distress among South African adults with adequate psychometric properties, good internal reliability, and good fit using a unidimensional model with correlated errors. This is consistent with previous work on the K-10 and K-6 in South Africa, Kenya and Ethiopia, and in other countries such as Australia. Future development of use of the K-10 in South Africa may include determining clinically significant cutoff values, addressing cultural elements, investigating the K-10 in different regions of South Africa and comparing the K-10 to other gold-standard diagnostic instruments to determine its clinical utility, sensitivity, and specificity in diverse populations. Further research is required to determine construct and criterion validity of the K-10 items incorporating local expressions of distress in the South African setting.

## Data Availability

This data will be made available through the United States’ National Institute of Mental Health Data Archive (NDA) through this website: https://nda.nih.gov/edit_collection.html?id=3805.

## Abbreviations

CIDI: Composite International Diagnostic Interview
CFA: Confirmatory factor analysis
CFI: Comparative fit index
DF: Degrees of freedom
DSM-5: Diagnostic and Statistical Manual of Mental Disorders-5
EFA: Exploratory factor analysis
GWAS: Genome-wide association study
IQR: Inter-quartile range
K-6: Kessler Psychological Distress Scale (6-item scale)
K-10: Kessler Psychological Distress Scale (10-item scale)
LMICs: Low- and middle-income countries
NeuroGAP: Neuropsychiatric genetics of African populations
PPV: Positive predictive value
RMSEA: Root mean square error of approximation
SD: Standard deviation
TLI: Tucker–Lewis index
